# An Optimized Method to Decellularize Human Trabecular Meshwork

**DOI:** 10.3390/bioengineering9050194

**Published:** 2022-04-30

**Authors:** Devon J. Crouch, Carl M. Sheridan, Julia G. Behnsen, Lucy A. Bosworth

**Affiliations:** 1 Department of Eye and Vision Science, Institute of Life Course and Medical Sciences, Faculty of Health and Life Sciences, University of Liverpool, Liverpool L7 8TX, UK; d.crouch@liverpool.ac.uk; 2 Department of Mechanical, Materials, and Aerospace Engineering, University of Liverpool, Liverpool L69 6GB, UK; julia.behnsen@liverpool.ac.uk

**Keywords:** decellularization, trabecular meshwork, eye, glaucoma, bioengineering, X-ray computed tomography, trypsin, ammonium hydroxide, lysing agent

## Abstract

Glaucoma is linked to raised intraocular pressure (IOP). The trabecular meshwork (TM) plays a major role in regulating IOP by enabling outflow of aqueous humor from the eye through its complex 3D structure. A lack of therapies targeting the dysfunctional TM highlights the need to develop biomimetic scaffolds that provide 3D in vitro models for glaucoma research or as implantable devices to regenerate TM tissue. To artificially mimic the TM’s structure, we assessed methods for its decellularization and outline an optimized protocol for cell removal and structural retention. Using bovine TM, we trialed 2 lysing agents—Trypsin (0.05% *v/v*) and Ammonium Hydroxide (NH_4_OH; 2% *v/v*). Twenty-four hours in Trypsin caused significant structural changes. Shorter exposure (2 h) reduced this disruption whilst decellularizing the tissue (dsDNA 26 ± 14 ng/mL (control 1970 ± 146 ng/mL)). In contrast, NH_4_OH lysed all cells (dsDNA 25 ± 21 ng/mL), and the TM structure remained intact. For human TM, 2% *v/v* NH_4_OH similarly removed cells (dsDNA 52 ± 4 ng/mL (control 1965 ± 233 ng/mL)), and light microscopy and SEM presented no structural damage. X-ray computed tomography enabled a novel 3D reconstruction of decellularized human TM and observation of the tissue’s intricate architecture. This study provides a new, validated method using NH_4_OH to decellularize delicate human TM without compromising tissue structure.

## 1. Introduction

Glaucoma is the leading cause of irreversible blindness worldwide, with a current incidence of 70 million that is expected to rise to ~112 million affected people by 2040 [1]. Glaucoma is an optic neuropathy that is associated with dysfunctional aqueous humor (AH) outflow from the anterior chamber of the eye, with the most prevalent form being primary open-angle glaucoma (POAG) [2,3,4,5]. This subset of glaucoma is defined by a normal, open iridocorneal angle but with an increased resistance to the conventional aqueous outflow pathway resulting in raised intraocular pressure (IOP) [6]. Elevated IOP is the major risk factor in causing retinal ganglion cell death, which results in the irreversible loss of vision [7]. The AH primarily drains from the eye via the trabecular meshwork (TM) into Schlemm’s canal [8]. To support this outflow, the TM, which bridges the iris to the peripheral cornea, has a complex 3D structure comprising collagenous beams, cells and extracellular matrix that changes with age and pathology to cause elevated IOP [9,10,11]. Elevated IOP is a consequence of reduced AH outflow and is caused by morphological and biochemical changes within the TM and the loss of TM cells [12]. Traditional anti-glaucoma treatments aim to reduce IOP by either reducing AH production or by increasing AH outflow, and both approaches are not without complications. To enhance AH outflow, various topical pharmacological agents or invasive surgical procedures that generate new channels for AH outflow have been utilized. Unfortunately, poor patient adherence diminishes the clinical efficacy of therapeutics [13], and postoperative complications, such as hypotony and fibrosis, can result in further surgical interventions being required [14]. Biomaterial devices provide an alternative to traditional methods for long-term maintenance of IOP [15]. These devices do not aim to replicate or regenerate the TM but offer channels to enable outflow of AH. Unfortunately, due to the stiffness of hard metals or plastics used in these types of devices, significant fibrosis can occur that leads to their eventual failure [16]. A tissue engineering approach that more specifically targets the TM, in terms of its recapitulation, may halt the development of POAG and regenerate the diseased tissue. Synthetic scaffolds fabricated from bioengineering techniques are able to closely imitate the structure of extracellular matrix (ECM) of human tissues [17]. This mimicry allows these scaffolds to confer spatial and topographical cues that prompt in vivo cell-like behavior in vitro [18]. Bioengineering could therefore be transformative in POAG research, where a three-dimensional (3D) biomimetic structure that exhibits TM-like in vivo characteristics could be employed as either an implantation device in glaucoma therapy or as a 3D in vitro model. A variety of fabrication techniques have been applied that all aim to mimic the TM and provide an alternative to current surgical devices [19,20,21]. Examples of these include freeze-casting to create a collagen-based biomaterial that is uniaxially aligned and with uniform porosity (pore size 10.3 ± 5.1 µm) [19]; photolithography to create scaffolds with a constant pore size (12 µm) and beam width (7.3 ± 0.1 µm) [20]; and electrospinning to produce fine fiber scaffolds (1.12 ± 0.01 µm) [21]. Though the structural uniformity observed in all of these scaffolds limits their biomimicry of this multi-zonal TM, which therefore results in reduced functionality of these biomaterials. The TM is known to comprise a hierarchical structure made up of three distinct filter regions: the uveal meshwork, corneoscleral meshwork and juxtacanalicular tissue. These regions differ anatomically from each other, consisting of connective tissue beams held in the form of lamellae or perforated sheets, which decrease in both beam width and pore size to form a natural porosity gradient through the tissue from the uveal meshwork to the juxtacanalicular [22]. Recapitulating the natural tissue structure in the form of biomimetic scaffolds is a recognized approach in biomaterial design [23,24], and fabrication of scaffolds that mimic the TM’s porosity gradient may provide the optimal artificial device to restore TM tissue function over time.

Multi-photon imaging [25], light [26] and electron [27] microscopy and histological evaluation [28] have all been employed to visualize cellularized human TM anatomy in two dimensions (2D). However, 2D images do not provide sufficient depth to allow thorough understanding and appreciation of this tissue’s fine-scale anatomical and three-dimensional (3D) hierarchical architecture. The presence of cells (and cellular debris) are also likely to obstruct the TM architecture during imaging. Tissue decellularization is a favorable method to improve 3D imaging of tissue structure. Porcine TM has been decellularized using a freeze–thaw approach with the aim to develop a cell transplantation therapy [29]. Though it should be noted that this type of physical method has been known to cause permanent damage to collagenous tissue [30]. Here we describe a chemical-based method for decellularization of human TM that does not impair its structure, visualized with high-resolution X-ray computed tomography to provide detailed observation of the TM’s 3D architecture without the hindrance of cells. To our knowledge, this is the first study that supports visualization of decellularized human TM in 3D. This is important for advancing the bioengineering design and development of synthetic biomimetic scaffolds to ensure accurate recapitulation of the TM architecture.

## 2. Materials and Methods

### 2.1. Decellularization of Bovine Trabecular Meshwork

#### 2.1.1. Sourcing Bovine Eyes

Bovine eyes (1–2 years old) were obtained from a local abattoir. Bovine eyes were retrieved and dissected 16 h post removal.

#### 2.1.2. Determining a Non-Destructive Decellularization Method Using Bovine Trabecular Meshwork

**Figure 1:**
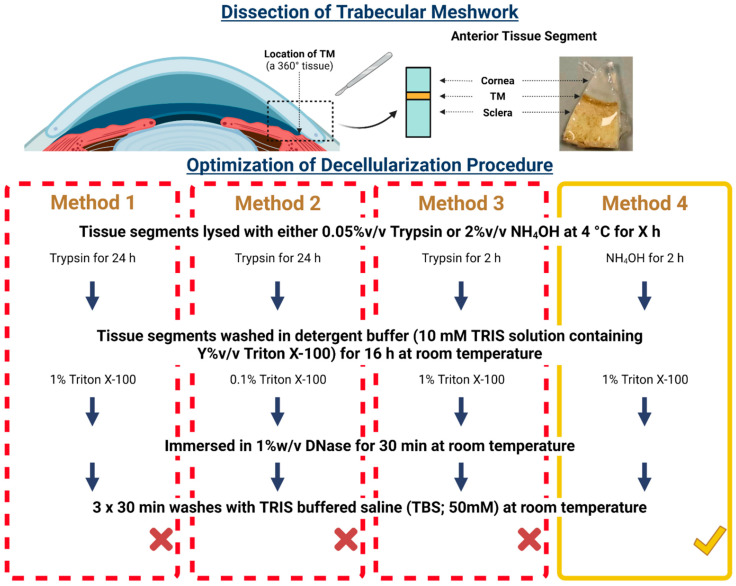
Schematic illustrating the different methods employed for complete decellularization of dissected bovine trabecular meshwork without compromising tissue structure. Method 4 was the optimal protocol for this tissue. Created with BioRender.com (accessed on 30 March 2022).

The anterior segment was dissected from bovine eyes, and the iris was removed to reveal the bovine TM (BTM), which was then carefully removed using forceps. The bovine eyes were cut into 8 equal segments (8 × 4 mm). From these, the TM was isolated from 6 of the 8 segments to give 6 BTM strips (3 mm) that were then placed into a 24-well plate (Corning, Flintshire, UK). The remaining 2 segments were used for histological evaluation. Different decellularization methods were employed with the aim of determining an optimal protocol; all times and concentrations applied are detailed in Figure 1. Overall, these decellularization processes are described as follows: BTM tissue strips were lysed using either 0.05% *v/v* Trypsin (Sigma-Aldrich, Gillingham, UK) or 2% *v/v* Ammonium Hydroxide (NH_4_OH; Fisher Scientific, Loughborough, UK) at 4 °C for X time (X = 2 or 24 h). After this time had elapsed, the lysing agent was removed, and all BTM strips were washed in detergent buffer (10 mM TRIS solution containing Triton X-100; Sigma-Aldrich, Gillingham, UK) at room temperature for 16 h, at Y concentration of Triton X-100 (Y = 0.01 or 1% *v/v*), to remove cellular fragments. BTM segments were then treated with 1% *w/v* DNase (Sigma-Aldrich, Gillingham, UK) for 30 min at room temperature to breakdown all remaining DNA within the tissue. Finally, the segments were washed for 30 min with 50 mM TRIS-buffered saline (TBS; Sigma-Aldrich, Gillingham, UK) thrice at room temperature. Tissues were subsequently fixed in appropriate fixative ready for DNA quantification or structural analysis.

#### 2.1.3. Immunocytochemical Analysis of BTM

Decellularized (dCELL) BTM strips were assessed for presence of cell nuclei via fluorescent imaging (n = 3) and compared to cellular BTM strips from the same tissue source. Bovine tissue strips were transferred to a 6-well plate (Corning, Flintshire, UK) and fixed in 10% Neutral Buffered Formalin (Sigma-Aldrich, Gillingham, UK) for 24 h at room temperature, followed by repeated washing with TBS (×3) prior to staining with 4′,6-diamidino-2-phenylindole (DAPI, 1:10,000; Fisher Scientific, Loughborough, UK), which was applied to fluorescently label cell nuclei. After staining, samples were imaged using a Zeiss Apotome microscope at ×10 and ×20 magnifications. Images were subsequently processed using Fiji ImageJ software (v2.0.0/1.53c).

#### 2.1.4. Imaging BTM with Scanning Electron Microscopy

Bovine tissue strips were imaged using scanning electron microscopy (SEM) to observe any architectural damage caused to the dCELL tissues’ structural matrix following decellularization compared to untreated cellular BTM (n = 3). Bovine tissue strips were fixed in 2.5% *v/v* Glutaraldehyde (TAAB Laboratories Equipment Ltd, Reading, UK) for 24 h at 4 °C. The strips were dehydrated in ascending concentrations of Ethanol (50–100% *v/v*; (Decon Laboratories Inc., King of Prussia, PA, USA)), critical point dried with Hexamethyldisilazane (HMDS; Sigma-Aldrich, Gillingham, UK) and mounted onto carbon-tabbed SEM stubs (Agar Scientific Ltd., Essex, UK). Mounted strips were Gold/Palladium (AuPd) sputter-coated to increase electrical conductivity and imaged using a HITACHI TM4000 Plus tabletop SEM at high vacuum with a 15 kV electron beam.

#### 2.1.5. Quantification of DNA Content for BTM

Bovine dCELL tissue strips were assessed for presence of residual double-stranded DNA (dsDNA) concentration compared to cellular TM controls (n = 3). A PicoGreen™ (Fisher Scientific, Loughborough, UK) assay was used to quantify dsDNA content of tissue samples. Cellular and dCELL strips were placed in a pre-made assay buffer (1% *v/v* Triton X-100, 0.5 mM Tris-HCL and 0.05 mM EDTA), vortexed for 20 s to ensure homogenization and frozen (−80 °C) to be preserved for future treatment. A 0.5% *v/v* PicoGreen™ solution was made in 0.5 mM Tris-HCL and 0.05 mM EDTA. Strips were subsequently thawed at room temperature, and 100 µL aliquots was dispensed into a black 96-well plate (Corning, Flintshire, UK). A total of 100 µL aliquots of PicoGreen™ working solution was added to each sample and gently mixed. Fluorescence was measured at 520 nm emission on a multi-plate reader (FLUOstar Omega; Isogen Life Science, Utrecht, Netherlands). Absorbance units were converted to DNA concentration using a standard curve.

#### 2.1.6. Histological Evaluation of BTM

Bovine dCELL tissue segments were fixed in 4% Paraformaldehyde (Sigma-Aldrich, Gillingham, UK) for 24 h. Tissue segments were then processed for embedding. Tissue segments were dehydrated in ascending concentrations of Ethanol, soaked in Xylene (Fisher Scientific, Loughborough, UK) and finally embedded in Paraffin wax (Leica Biosystems, Milton Keynes, UK). Tissue segments were sectioned (4.5 µm slices) and stained with Hematoxylin and Eosin (H&E; (Leica Biosystems, Milton Keynes, UK)) to enable observation of cell nuclei and any damage to the tissue structure. Comparison was made to cellular BTM.

#### 2.1.7. Statistical Analysis of BTM

Statistical analysis was performed using GraphPad prism v9.1.0. A one-way repeated measure analysis (ANOVA) with Tukey’s multiple comparisons test for pairwise comparison of dsDNA concentration was assessed, and statistical significance was set at *p* < 0.05.

### 2.2. Decellularization of the Human Trabecular Meshwork

#### 2.2.1. Sourcing Human Eyes

Donated human cadaver eyes (81, 88 and 88 years old) were acquired from the Liverpool Research Eye Bank. All donated eyes were free from glaucoma and other eye conditions at time of death. The study was conducted in accordance with the Declaration of Helsinki, with prior ethics approval from the National Research Ethics Service Committee (reference: 8335).

#### 2.2.2. Decellularization of the Human Trabecular Meshwork Using the Optimized Non-Destructive Method

The anterior segment was dissected from human cadaver eyes and cut into 10 equal segments (4 × 2 mm). Anterior tissue segments were used in human decellularization studies instead of isolating the TM as conducted in bovine analysis, due to their smaller tissue size and difficulty in handling. Anterior segments consisted of the TM, cornea, iris and sclera. The iris was removed from all but 2 tissue segments to reveal the human TM (HTM). Tissue segments with iris intact were used for histological analysis to allow more accurate orientation of the tissue and to permit visualization of the iridocorneal angle in greater detail. All tissue segments were placed into a 24-well plate. Anterior segments were treated in accordance with Method 4 detailed in Figure 1, which involved a 2 h NH_4_OH (2% *v/v*) treatment at 4 °C, a 16 h detergent wash (10 mM TRIS containing 1% *v/v* Triton X-100), a 30 min immersion in DNase (1% *w/v*) and finally 3 × 30 min washes in 50 mM TBS. After treatment was concluded, cellular and dCELL HTM segments were assessed by dsDNA quantification as described in Section 2.1.5, histological evaluation as described in (Section 2.1.6) and SEM analysis as described in (Section 2.1.4).

#### 2.2.3. X-ray Computed Tomography Imaging of HTM

X-ray computed tomography (X-CT) was used to visualize dCELL and cellular HTM tissues in 3D. Tissue segments used for SEM analysis were re-used for X-CT scanning.

X-CT scans were acquired using a Zeiss Xradia Versa 620 instrument. SEM stubs with mounted HTM were placed in the sample holder. The 4× objective was selected, and source and detector distances were set to achieve a voxel size of 1.08 µm, which resulted in a 1.1 mm × 1.1 mm field of view (detector binning 2). A source accelerating voltage of 60 kV at 6.5 W was applied. A beam filter was not used. For each scan, 3201 projections were acquired over 360° with an exposure time of 3 s per projection. The projection data were reconstructed using Zeiss proprietary Reconstructor Scout-and-Scan, v16.1.13038, which employs a cone beam Feldkamp–Davis–Kress algorithm. Three-dimensional images of the reconstructed data were created with Drishti 2.7 [31].

#### 2.2.4. Statistical Analysis of HTM

Statistical analysis was performed using GraphPad prism v9.1.0. A paired *t*-test (n = 3) of dsDNA concentration was conducted (comparison to untreated, cellular HTM); statistical significance was set at *p* < 0.05.

## 3. Results

### 3.1. Determining an Optimal Method for BTM Decellularization

#### 3.1.1. Immunocytochemical Evaluation of BTM

**Figure 2:**
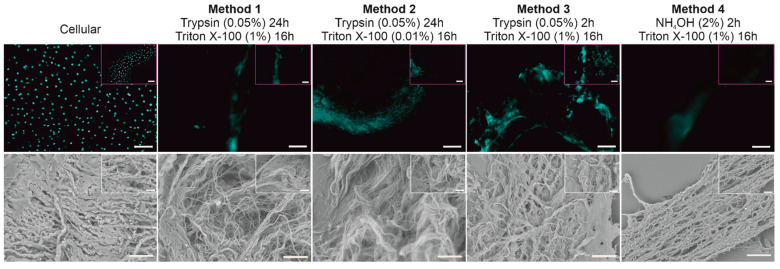
Representative apotome fluorescent images (**Top**) and scanning electron micrographs (**Bottom**) of bovine trabecular meshwork treated with four different decellularization protocols with comparison to cellular control. Fluorescent staining of cell nucleus with DAPI (blue). (**Top**: magnification ×10, scale bar = 100 µm; inset magnification ×20, scale bar = 50 µm; **Bottom**: magnification ×1000, scale bar = 50 µm; inset magnification ×2500, scale bar = 20 µm).

Four different decellularization methods were trialed to determine an optimal protocol capable of removing all native cells from the BTM without affecting its structure. DAPI staining indicated the presence of nuclei in cellular BTM but not in dCELL BTM for any of the protocols trialed; here only background auto-fluorescence was observed (Figure 2, Top).

#### 3.1.2. Structural Evaluation of BTM Using SEM

SEM imaging was conducted on the tissue samples to observe if any structural damage had occurred following the chemical/enzymatic treatments employed (Figure 2, Bottom). Methods involving Trypsin treatment for 24 h resulted in damage to the BTM tissue with the structural matrix appearing unraveled and pushed apart (Methods 1 and 2). When a shorter exposure time of 2 h was implemented (Method 3), the degree of disruption to the BTM architecture was notably reduced, with the structural matrix still resembling the control tissue. Use of NH_4_OH-treated BTM (Method 4) appeared to cause no structural damage to the tissue, which remained visually intact and resembled the cellular BTM tissue.

#### 3.1.3. Quantifying DNA Content of BTM

**Figure 3:**
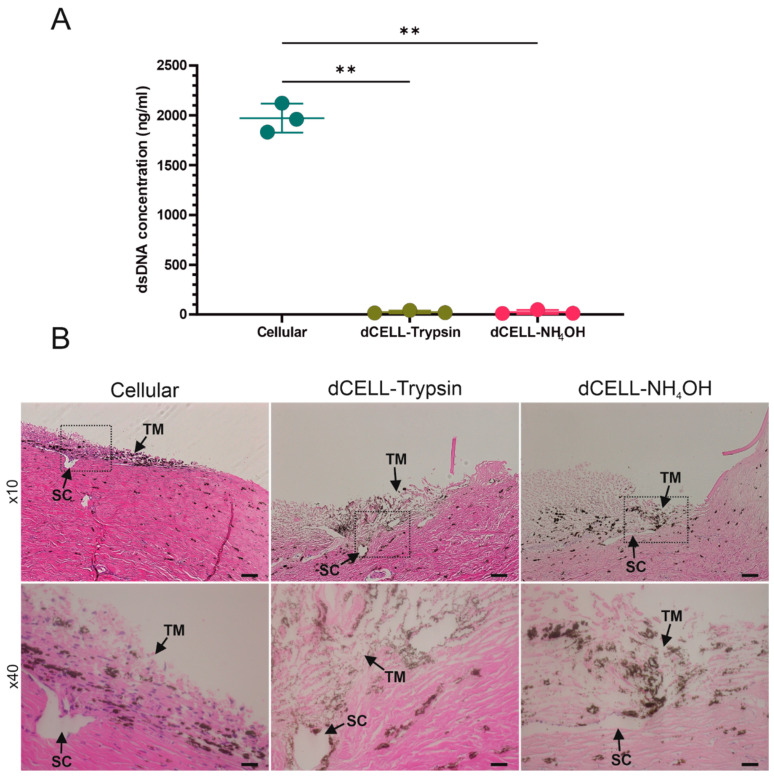
(**A**) Comparison of double-strand DNA concentration in cellular and decellularized (dCELL) bovine trabecular meshwork using either Trypsin (0.05%, 2 h) or Ammonium Hydroxide (NH_4_OH; 2%, 2 h). One-way repeated measures ANOVA with Tukey’s multiple comparisons (n = 3), significance for *p* < 0.05, ** represents *p* < 0.01. (**B**) H&E-stained images of cellular and dCELL bovine trabecular meshwork. Trabecular meshwork (TM) and Schlemm’s canal (SC) located by black arrows. Dashed black box in ×10 magnification images indicate region of interest for ×40 magnification images. BTM structure contains regions of pigmentation. Scale bars = 50 µm (×10) and 20 µm (×40).

Decellularization methods taken forward for further data analysis included Trypsin (dCELL–Trypsin (Method 3)) and NH_4_OH (dCELL–NH_4_OH (Method 4)) treatments for 2 h with 16 h detergent washing in a 1% *v/v* Triton X-100 solution. Cellular remnants in dCELL segments were quantified using a PicoGreen™ assay and compared to cellular counterparts (n = 3; Figure 3A). Decellularized BTM saw a significant reduction in dsDNA concentration after treatment for both Trypsin (26 ± 14 ng/mL, *p* = 0.004) and NH_4_OH (25 ± 21 ng/mL, *p* = 0.004) when compared to cellular BTM (1970 ± 146 ng/mL).

#### 3.1.4. Histological Evaluation of BTM

Histological evaluation of the BTM was conducted using H&E to observe cell presence in both cellular and dCELL samples to further validate decellularization. Immunohistochemical staining revealed visible cell nuclei in cellular BTM, as opposed to both dCELL samples, which appeared to be fully decellularized (Figure 3B).

### 3.2. Decellularization of HTM Using Optimized Method

Of the protocols trialed, Method 4 was the only protocol that successfully removed all cells and did not disrupt the BTM structure. This optimized method was subsequently applied to human TM.

#### 3.2.1. Quantifying DNA Content of HTM (Anterior Tissue Segment)

**Figure 4:**
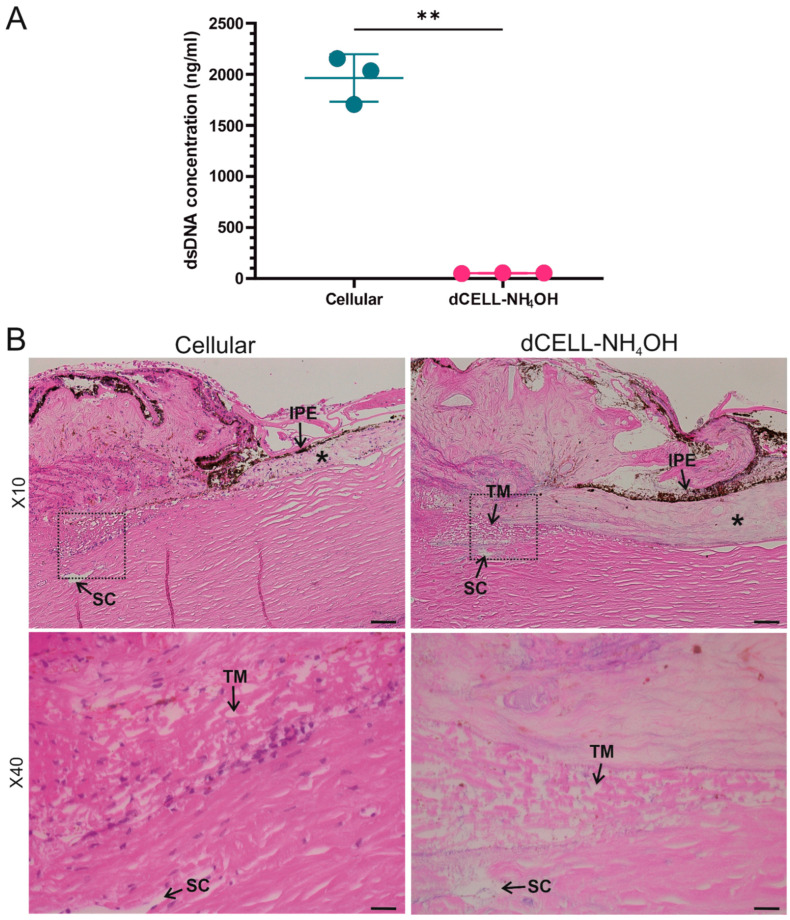
(**A**) Comparison of double-strand DNA concentration in cellular and decellularized human trabecular meshwork using Ammonium Hydroxide (NH_4_OH, 2%, 2 h). Paired *t*-test (n = 3), significance for *p* < 0.05, ** represents *p* < 0.01. (**B**) Representative H&E-stained images of cellular and dCELL–NH_4_OH human trabecular meshwork. Trabecular meshwork (TM) and Schlemm’s canal (SC) located by black arrows. * indicates the iris. Dashed black box in ×10 magnification images indicate region of interest for ×40 magnification images. Pigmentation in tissue structure is the iris pigmented epithelium (IPE), located by black arrows. Scale bars = 50 µm (×10) and 20 µm (×40).

The presence of dsDNA for HTM following decellularization in NH_4_OH (dCELL–NH_4_OH (Method 4)) was evaluated and directly compared to cellular counterparts (n = 3; Figure 4A). A significant reduction of dsDNA concentration was observed in the dCELL–NH_4_OH HTM (52 ± 4 ng/mL; *p* = 0.005) when compared to the cellular tissue (1965 ± 233 ng/mL).

#### 3.2.2. Histological Evaluation of HTM (Anterior Tissue Segment)

Histological evaluation using H&E was conducted to further validate complete decellularization of treated HTM samples by observing presence of cell nuclei (or lack thereof) when compared to cellular HTM. There was no evidence of cellular material observed in H&E-stained sections for tissues subjected to dCELL–NH_4_OH. Visible cell nuclei were present in the paired cellular controls (Figure 4B).

#### 3.2.3. Structural Evaluation of HTM Using SEM

**Figure 5:**
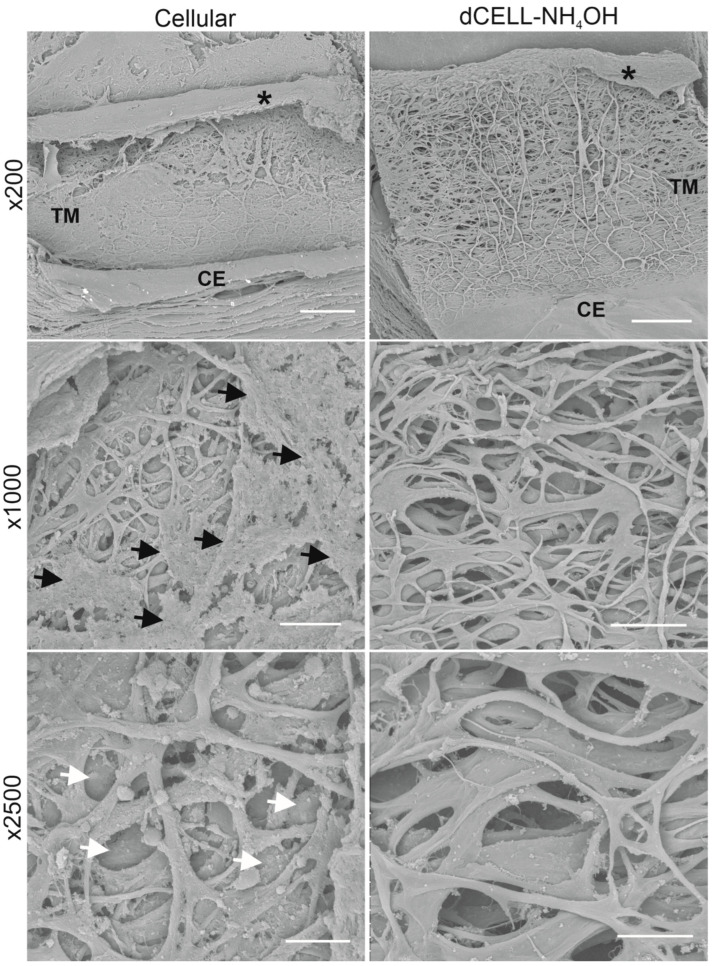
Representative scanning electron micrographs for cellular and dCELL–NH_4_OH human trabecular meshwork (magnifications ×200, ×1000 and ×2500). Black arrows indicate presence of cells or cellular debris within the tissue. White arrows indicate blocked pores within tissue. Trabecular meshwork (TM) and corneal endothelium (CE) labelled in ×200 image. * represents where the iris was removed to allow visualization of the TM. Scale bars = 200 µm (×200); 50 µm (×1000); 20 µm (×2500).

SEM analysis was conducted to visualize the HTM architecture and observe if any structural damage had occurred following NH_4_OH treatment. No evidence of structural impairment was observed with the dCELL–NH_4_OH HTM, which presented a likeness to cellular HTM (Figure 5). Though cellular HTM tissues did present a couple of notable differences; including a clear presence of cells (or cellular debris) visible at ×200 and ×1000 magnifications (black arrows), and imaging at ×2500 demonstrated regions of pores appearing blocked (white arrows), which obstructed the visual depth within the tissue. In comparison, the dCELL–NH_4_OH HTM presented an unobstructed and fully porous structure even when viewing at low magnification (×200). At ×2500, an open-network structure with depth through the layers was clearly observable. Minimal foreign matter was present on the surface of the fibrous structure, which was likely to be fragments of cellular debris remaining on the tissues’ matrix post treatment.

#### 3.2.4. X-CT Imaging and 3-Dimensional Reconstruction of the HTM

**Figure 6:**
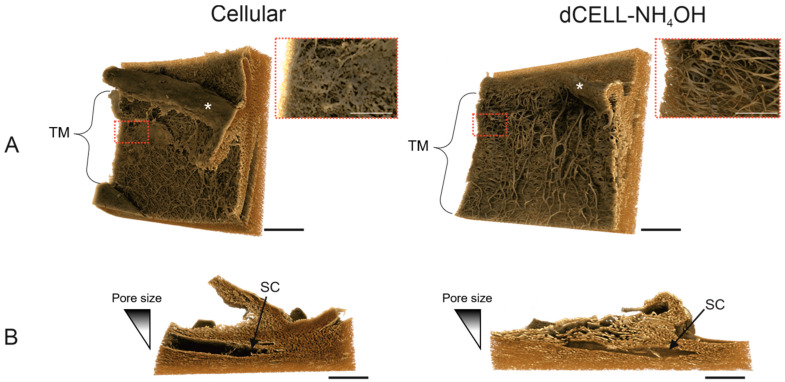
X-ray computed tomography images of cellular and dCELL–NH_4_OH human trabecular meshwork shown in (**A**) front-on and (**B**) cross-sectional orientations. Red dashed boxes indicate region of interest for zoomed-in views. Trabecular meshwork (TM) and Schlemm’s canal (SC) indicated by curly bracket and black arrow, respectively. * represents where the iris was removed to allow visualization of the TM. ((**A**): Scale bar = 200 µm; inset scale bar = 50 µm and (**B**): Scale bar = 200 µm).

Using the same tissue samples for SEM imaging, cellular and dCELL HTM segments were scanned using X-CT, and 3D images were reconstructed (Figure 6). The cellular HTM sample had a visible porous structure that was clearly obscured by cells and possible cellular debris (Figure 6A). The zoomed inset image displayed definitive evidence of this with visual evidence of cellular presence hindering the ability to observe deeper within the 3D tissue. Comparatively, imaging of the dCELL–NH_4_OH HTM revealed a clear view of structural beams and pores devoid of cells spanning across the tissue segment (Figure 6A). A zoomed-in image of the dCELL structure is displayed in the inset, where the depth of the fibrous network can be better appreciated. Cross-sectional views of dCELL–NH_4_OH HTM further revealed a clear gradient of porosity within the tissue, becoming more compact as the fibrous network approached Schlemm’s canal (SC) (Figure 6B). This observation was less apparent in the cellular tissue, which appeared tightly compact throughout.

## 4. Discussion

The aim of this study was to optimize a decellularization method that removes native cells from the HTM without compromising its structure. Current decellularization procedures can be grouped into three categories: physical, chemical and biological [32]. Physical methods often involve freeze–thaw, direct pressure, mechanical agitation or sonication [33]. Chemical agents, such as acid/bases, detergents (non-ionic, zwitterionic or ionic) and hypo-/hypertonic solutions, are perfused through the target tissue to lyse cells from the ECM [34]. Biological agents, such as nuclease, decellularize the host tissue via enzymatic breakdown [35]. However, for successful decellularization, a mixture of these techniques is often combined [36]. Decellularization of tissues is routinely applied for harvesting the structural and functional proteins that comprise the ECM [32]. These acellular biological scaffolds can then be used to either observe the complex ECM structure, recellularized with host’s cells for implantation or pulverized into ECM powder for application in tissue engineering [37,38]. The intended use of the decellularized tissue heavily influences the technique used to remove cells from the tissue, for example, production of ECM powder requires pulverization in a grinding mill and, therefore, obliterates the tissue structure [39]. Furthermore, decellularized tissues that are to be implanted into the non-native host need to ensure effective removal of cellular components to minimize the immunogenicity of these allogeneic or xenogeneic biological scaffolds [40]. Whereas if the goal is to observe the structure of the tissue void of cells, the method still requires effective removal of the majority of cells and their remnants, but it is not necessary that dsDNA content is below the recommended threshold of 50 ng/mL [41]. While decellularization techniques involving chemical agents have been used for other ocular tissues such as the conjunctiva [42], cornea [43] and retina [44], there is no such method described for the TM to date. Although, a freeze–thaw method has been previously applied to decellularize porcine TM [29]. Whilst all cells were successfully removed, there is a risk the tissue structure could be adversely affected, as it is well known that this type of physical decellularization technique can affect collagen structure [30]. This would be detrimental to our goal as it is imperative the TM structure is retained for subsequent biomimicry. Therefore, we concentrated on using chemical agents only to decellularize the human TM and hence reduce the risk of causing structural damage. We believe this is the first chemical-based decellularization protocol optimized for HTM tissue to be reported.

The protocol was first optimized using a non-primate mammalian species. Whilst non-primate TM anatomy has a reticular organization (i.e., net-like structure) compared to primate TM anatomy, which is trabecular (i.e., porous fiber network), the ethical and economic barriers in sourcing other primate species, such as monkeys, renders non-primates the best option for the first part of our study [45]. TMs from bovine eyes were used due to their low cost, high availability and ease of dissection [46]. Four decellularization methods—which used either Trypsin or NH_4_OH as the main lysing agent—were employed to void BTM of cells (Figure 1). Trypsin is known to remove cells from the tissue by cleavage of the cell cytoskeleton from the ECM through hydrolysis of peptide bonds [47], and as an alkaline agent, NH_4_OH causes disruption to the cell membrane leading to osmotic swelling and cell lysis [48]. The main difference among the four methods trialed for decellularization of BTM involved adjusting the time associated with the lysing agent (24 h or 2 h) to assess the ability to remove cells whilst limiting damage to the tissue’s structure.

Immunocytochemical staining with DAPI was used to observe the presence of cell nuclei as the fluorescent stain strongly binds to adenine–thymine-rich regions in DNA [49]. Furthermore, a lack of visible cellular material in tissue sections has been proposed as one of the criteria for successful decellularization, along with <50 ng/mg dsDNA and <200 base pairs of DNA fragment length [41]. Cells were clearly visible in the cellular BTM tissue (Figure 2, Top). In contrast, all treated tissues had no evidence of cell nuclei with only background autofluorescence observed, demonstrating cells had been effectively removed. Similarly, SEM was used to provide visual assessment of the tissues following treatment (Figure 2, Bottom). Although successful decellularization occurred using Trypsin (Method 1), the remaining ECM structure of the BTM tissue became unraveled post treatment and no longer resembled the cellular BTM tissue. To counteract this disruption to the ECM, the concentration of the non-ionic detergent was reduced from 1% *v/v* to 0.01% *v/v* (Method 2). Whilst decellularization was successful, the reduction in detergent concentration still caused disruption to the ECM structure, which also appeared as an unraveled fibrous network (Figure 2, Method 2). This suggests that the detergent, Triton X-100, was not the cause of BTM destruction. Method 3 trialed a reduction in Trypsin lysis treatment time from 24 h to 2 h to observe if this would reduce the structural damage. This shorter treatment time did appear to reduce the extent of disruption observed in the dCELL BTM, but viewing at higher magnification (Figure 2, Method 3, inset) revealed evidence of loose fibers. Use of Trypsin as a lysing agent demonstrated its utility for successful cell removal, but damage to the tissue structure renders it too harmful for the purpose of later tissue mimicry. Other studies in the literature also report loss of biomolecules, such as glycosaminoglycans, reduced mechanical properties and prolonged exposure disrupting the ECM of the tissue when using Trypsin as the lysing agent [50,51,52]. NH_4_OH was therefore introduced in Method 4 as a replacement lysis agent as reports of surrounding tissue structures and mechanical properties remaining unharmed have been reported [53]. Furthermore, NH_4_OH has been used in TM cell-derived matrix studies to successfully decellularize deposited extracellular matrix without causing physical damage [54,55,56]. An analysis of NH_4_OH-treated BTMs demonstrated successful removal of cells and retention of tissue structure when compared to the cellular BTM architecture (Figure 2, Method 4). Methods 3 and 4 were both successful in removing cells from the BTM after 2 h treatment with minimal to no disruption of the structure with Trypsin (dCELL–Trypsin) and NH_4_OH (dCELL-NH_4_OH), respectively. Hence, investigation into the decellularization of BTM using other common lysing agents—such as sodium dodecylsulfate—was not required. Furthermore, due to the success observed with Methods 3 and 4, comparisons between these two methods were subsequently conducted to evaluate their suitability to decellularize the BTM without compromising its structure. Here, dCELL BTM strips and segments were assessed by double-stranded DNA (dsDNA) content and histological evaluation, respectively. The presence of residual dsDNA in dCELL tissue strips were quantified by a PicoGreen™ assay and compared to paired cellular controls (Figure 3A). Complete decellularization was observed in both dCELL–Trypsin (26 ± 14 ng/mL) and dCELL–NH_4_OH BTM tissue (25 ± 21 ng/mL), where remnants of dsDNA in the tissue should be less than 50 ng/mL to be classed as fully decellularized [41]. Both Methods 3 and 4 yielded a significant reduction (*p* < 0.004) in dsDNA compared to their cellular counterparts (1972 ± 146 ng/mL). Histological analysis using H&E staining on BTM segments validated the presence (or lack) of cell nuclei with complete decellularization in treated tissues compared to cellular BTM observed.

From these data, the optimized method for successful decellularization of BTM without affecting its structure is to lyse the tissue in NH_4_OH for 2 h with a 16 h detergent wash in Triton X-100 (Figure 1, Method 4). This is because the SEM images of Method 3 (Figure 2, Bottom) revealed some disruption to the structure of the BTM, whereas Method 4 fully decellularized the BTM without affecting the integrity of its architecture. This optimized method was subsequently applied to HTM. To validate the transfer of this protocol to HTM tissue (held within a larger segment of the anterior tissues due to difficulties in handling completely isolated TM), we repeated our analyses in terms of DNA quantification, histology and SEM and further explored the tissue structure using X-CT.

Quantification of dsDNA revealed treatment with NH_4_OH successfully decellularized HTM tissue for all three donors (52 ± 4 ng/mL) compared to cellular HTM (1965 ± 233 ng/mL (*p* = 0.005)) (Figure 4A). Whilst this dsDNA content is slightly higher than the suggested threshold of 50 ng/mL, a lack of cell nuclei present in the dCELL–NH_4_OH tissue after histological evaluation (Figure 4B) suggests the human tissue was fully decellularized [41]. This slightly higher dsDNA concentration is likely a consequence of using an anterior tissue segment containing TM, sclera and cornea, as opposed to solely isolated TM used in the previous bovine studies. Histological evaluation confirmed complete decellularization of the HTM post treatment, though this elevated dsDNA content is of little concern for our aim of visualising HTM architecture.

Low magnifications (×10) used for histological evaluation render it an unreliable method for determining disruption to the HTM tissue post treatment. Therefore, SEM imaging at considerably higher magnifications was conducted to observe the structural integrity of the dCELL–NH_4_OH HTM compared to cellular. SEM analysis revealed flat beams with no obvious breaks in the fibrous structure for dCELL–NH_4_OH HTM, which was comparable to the paired cellular controls (Figure 5). These observations further validate this method for the preservation of HTM’s structure. Yet, subtle differences were evident: ×1000 magnification revealed the presence of cells (or cellular debris) (indicated by black arrows), and ×2500 magnification exposed pores between the beams to be blocked (white arrows). These blockages are likely to be caused by cells obscuring the pores as the donors were known not to have glaucoma, and a comparison to decellularized counterparts demonstrated an open-porous network free from blockages.

SEM is an ideal technique for monitoring the structural integrity of the HTM post-decellularization but, being a surface imaging technique, the depth of the tissue’s porosity cannot be fully gauged. X-ray computed tomography (X-CT) was subsequently applied to allow visualization of tissue structural integrity for both cellular and dCELL–NH_4_OH HTM in 3D. Whilst X-CT has been previously used in HTM research to visualize the outflow pathway of the eye [57] (specifically Schlemm’s canal and collector channels) and insertion of a second generation iStent into Schlemm’s canal [58], it—to our knowledge—has never been used to visualize the HTM’s intricate 3D structure. Furthermore, a smaller voxel size of 1.08 µm was achieved using the Xradia Versa 620 instrument, generating images of considerably higher resolution when compared to the 2 µm and 5 µm used in these reported studies. Images shown in Figure 6 are X-CT scans of the same tissue segments imaged using SEM. In Figure 6A, a front-on view of the porous TM is presented for both the cellular and dCELL–NH_4_OH HTM samples. A more enhanced, close-up view of the structure revealed the cellular HTM fiber structure to be partially obstructed by cells (inset). Conversely, X-CT images of dCELL–NH_4_OH enabled a clear depth of the HTM tissue void of cells to be clearly observed. Inclusion of this z-plane demonstrates the added advantage of using this technique over 2D SEM imaging to gain a complete understanding of the tissue’s 3D architecture. In addition, cross-sectional images of the dCELL–NH_4_OH obtained from X-CT imaging (Figure 6B) expose the gradient of porosity—a key characteristic of HTM—in greater detail than observed in histological images in Figure 4B. This gradient is indistinguishable in the cellular HTM likely due to the presence of cells blocking the open pores, which further validates the need for complete tissue decellularization to fully appreciate the HTM ultrastructure without interference.

This study determined the optimal method for complete decellularization of HTM without compromising its tissue structure. For HTM, this method involves a 2 h treatment with NH_4_OH (2% *v/v*), a 16 h wash with Triton X-100 (1% *v/v*) detergent buffer, a 30 min immersion in DNase (1% *w/v*) and 3 × 30 min washes in TBS (50 mM) and was confirmed using multiple techniques including, dsDNA quantification, histology, SEM and X-CT scanning. Novel imaging of this intricate tissue in 3D will enable key information regarding change in pore size, porosity and fiber/beam diameters to be extracted and used to develop future biomimetic synthetic scaffolds to advance glaucoma research through advanced 3D in vitro models that recapitulate the tissue and implantables for TM regeneration in vivo.

## 5. Conclusions

This methods paper describes the optimal protocol for successfully decellularizing BTM without compromising its tissue architecture. Whilst effective in removing all cellular content, Trypsin was demonstrated to be an unsuitable treatment and should be avoided if retention of delicate tissue structure is required. NH_4_OH treatment, however, successfully lysed cells whilst maintaining tissue integrity. This method was successfully transferred to HTM tissues, where cells were removed, and the tissue structure also remained unaffected. This method validation demonstrates NH_4_OH as the optimal treatment for tissue decellularization and preservation of the human TM structure. X-CT scanning enabled decellularized HTM architecture to be clearly visualized in 3D, and information from these images can be used to provide detailed information and act as a template for the future design of scaffolds that recapitulate the TM structure.

## Data Availability

Zeiss original format.txm files can be found with the following DOI: https://doi.org/10.17638/datacat.liverpool.ac.uk/1658 (accessed on 7 April 2022). All other data presented in this study are available on request from the corresponding authors.
